# Adiponectin signaling regulates urinary bladder function by blunting smooth muscle purinergic contractility

**DOI:** 10.1172/jci.insight.188780

**Published:** 2025-02-24

**Authors:** Zhaobo Luo, Ali Wu, Simon Robson, Seth L. Alper, Weiqun Yu

**Affiliations:** 1 Division of Nephrology,; 2 Department of Anesthesia, Beth Israel Deaconess Medical Center, Boston, Massachusetts, USA.; 3 Department of Anesthesia, Harvard Medical School, Boston, Massachusetts, USA.; 4 Department of Medicine, Beth Israel Deaconess Medical Center, Boston, Massachusetts, USA.; 5 Department of Medicine, Harvard Medical School, Boston, Massachusetts, USA.

**Keywords:** Endocrinology, Metabolism, Adipose tissue, Muscle, Urology

## Abstract

Lower urinary tract symptoms (LUTS) affect approximately 50% of the population over 40 years of age and are strongly associated with obesity and metabolic syndrome. Adipose tissue plays a key role in obesity/metabolic syndrome by releasing adipokines that regulate systemic energy/lipid metabolism, insulin resistance, and inflammation. Adiponectin (ADPN), the most abundant adipokine, modulates energy/metabolism homeostasis through its insulin-sensitizing and antiinflammatory effects. Human plasma ADPN levels are inversely associated with obesity and diabetes. To the best of our knowledge, the role of adipokines such as ADPN in the LUTS associated with obesity/metabolic syndrome remains unknown. We have tested such a possible role in a global ADPN-knockout (*Adpn^–/–^*) mouse model. *Adpn^–/–^* mice exhibited increased voiding frequency, small voids, and reduced bladder smooth muscle (BSM) contractility, with absence of purinergic contraction. Molecular examination indicated significantly altered metabolic and purinergic pathways. The ADPN receptor agonist AdipoRon was found to abolish acute BSM contraction. Intriguingly, both AMPK activators and inhibitors also abolished BSM purinergic contraction. These data indicate the important contribution of what we believe is a novel ADPN signaling pathway to the regulation of BSM contractility. Dysregulation of this ADPN signaling pathway might be an important mechanism leading to LUTS associated with obesity/metabolic syndrome.

## Abstract 2

Dysregulation of fat-released hormones can affect bladder smooth muscle contractility and lead to bladder dysfunction in populations with obesity and metabolic symptoms.

## Graphical abstract

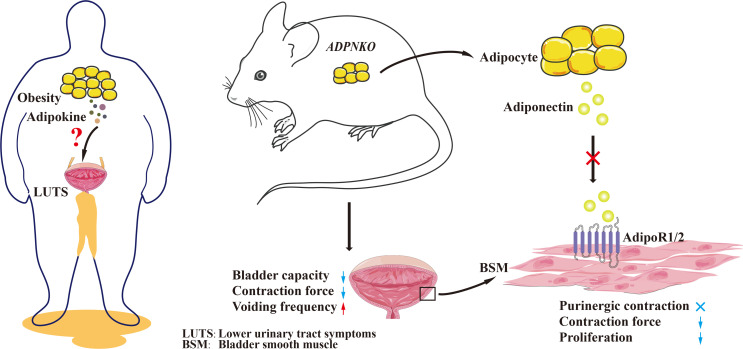

## Introduction

The fluctuating volume of the urinary bladder reflects cycles of filling and voiding, during which urothelial cells (UCs) undergo reversible morphological transitions between goblet-like and squamous cells. This process is driven by membrane exocytosis/endocytosis (1–3), enabling UCs to accommodate fluctuations in urine volume (storage function) and to release neurotransmitters such as ATP for micturition (voiding function) (4). Bladder smooth muscle (BSM) cells are relaxed for elongation during filling and activated to contract during voiding. Regulation of contraction and relaxation involves muscarinic receptors M2 and M3, purinergic receptors such as P2X1, β3 adrenergic receptors, and adenosine A2b receptors. Dysfunction of UCs and BSM cells can produce lower urinary tract symptoms (LUTS), which affect approximately 50% of the population that is 40 years of age or older (5, 6). The symptoms include urinary incontinence (UI), urgency, overactive bladder (OAB), and underactive bladder (UAB), and their underlying mechanisms are incompletely understood.

Obesity affects over one-third of the US population. Visceral obesity, in particular, is a hallmark of metabolic syndrome and aging (7–9). LUTS is also strongly associated with obesity, metabolic syndrome, and aging (10–13). A direct link between obesity/metabolic syndrome and LUTS is convincingly suggested by the beneficial effects of bariatric surgery, caloric restriction, and physical exercise on both UI and OAB (10, 11, 13–15). However, the molecular mechanisms by which obesity/metabolic syndrome predisposes to LUTS remain poorly understood. Adipose tissue (fat), in addition to functioning as an energy storage site, is also an important endocrine organ that releases physiologically active, circulating adipokines, including leptin, tumor necrosis factor α (TNF-α), adipsin, plasminogen activator inhibitor-1, interleukin 6, resistin, monocyte chemotactic protein-1, adiponectin (ADPN), and others. These adipokines are important regulators of energy and lipid metabolism, insulin resistance, inflammation, and many other cellular functions (7–9, 16). The relationship between adipokines and LUTS is unclear.

We have recently reported that mice with smooth muscle–specific deletion of the insulin receptor (*SMIR^–/–^*) had abnormal bladder phenotypes, including urinary frequency and small voids, and decreased contractility of BSM. The study suggested that dysregulated insulin signaling or insulin resistance in local bladder tissue might be important to the development of diabetic bladder dysfunction (DBD) (17). Interestingly, we observed downregulation of ADPN expression in the bladders of *SMIR^–/–^* mice (17). As ADPN is predominantly released by adipose tissue, we investigated bladder tissue expression of ADPN. Our immunofluorescence localization data indicated the expression of ADPN in BSM cells (17). These discoveries suggest a potential ADPN signaling mechanism contributing to the regulation of bladder function.

ADPN is the most abundant adipokine secreted mainly by adipose tissue (18, 19). ADPN levels decrease with progression of obesity/metabolic syndrome and with age, whereas elevated levels are generally protective (20–23). Elevated ADPN levels exert insulin-sensitizing, antidiabetic, antiinflammatory, and antiatherogenic effects. Global ADPN-knockout mice fed a high-fat diet develop exacerbated insulin resistance and metabolic syndrome, with shortened lifespan (24–26). In contrast, transgenic mice with elevated circulating ADPN levels exhibit dramatically improved systemic insulin sensitivity, reduced age-related tissue inflammation, and prolonged lifespan (25). ADPN gene mutations and polymorphisms in humans are closely associated with low circulating ADPN levels, insulin resistance, and type II diabetes (27, 28). These studies suggest ADPN as a key adipokine modulating progression of metabolic syndrome. Whether and how adiponectin signaling regulates bladder function has remained unclear. However, in a mouse model with a 56% increase in plasma ADPN, BSM cells exhibited altered Ca^2+^ sensitivity, supporting a functional role of ADPN signaling in BSM (29).

The association between LUTS and obesity/metabolic syndrome, and the strong correlation between adipokine dysregulation and obesity/metabolic syndrome in both patients and animal models prompted our hypothesis that dysregulated adipokine signaling is a major contributor to the pathogenesis of LUTS. We have tested our hypothesis in this study using a global ADPN-knockout (*Adpn^–/–^*) mouse model.

## Results

### Adpn^–/–^ mice exhibit altered voiding phenotype and urodynamics.

**Figure 1:**
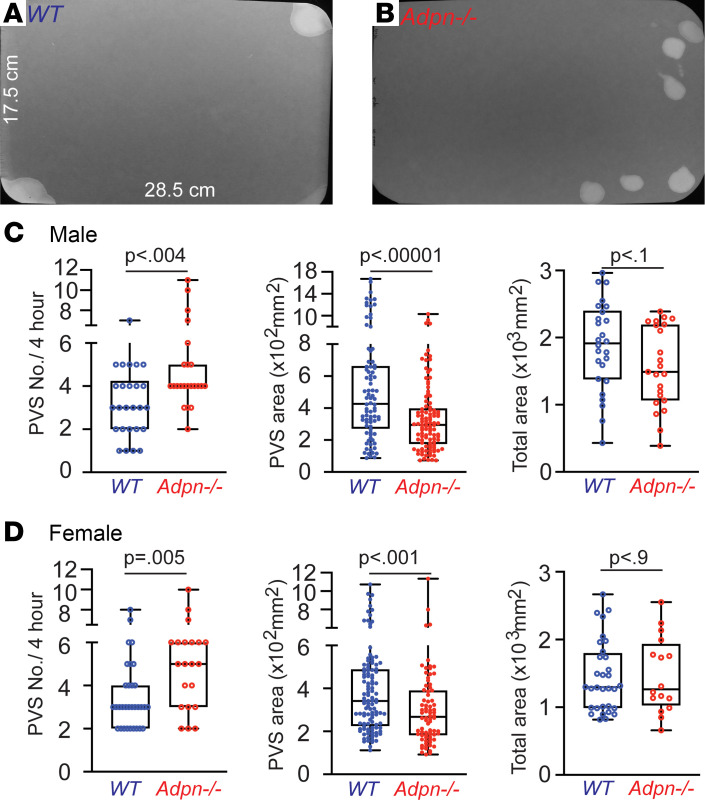
*Adpn^–/–^* mice exhibit increased voiding frequency and small voids. Representative VSA filter images are shown for (**A**) WT (*n* = 26 male, *n* = 32 female) and (**B**) *Adpn^–/–^* (*n* = 23 male, *n* = 21 female) mice. (**C** and **D**) Primary voiding spot (PVS) counts, defined as voiding spots larger than 80 mm^2^, for males (**C**) and females (**D**). Data are plotted in box (75% of the data) and whisker format (minimum to maximum), with centerline as median value. Student’s *t* test, with *P* values above bars.

**Figure 2:**
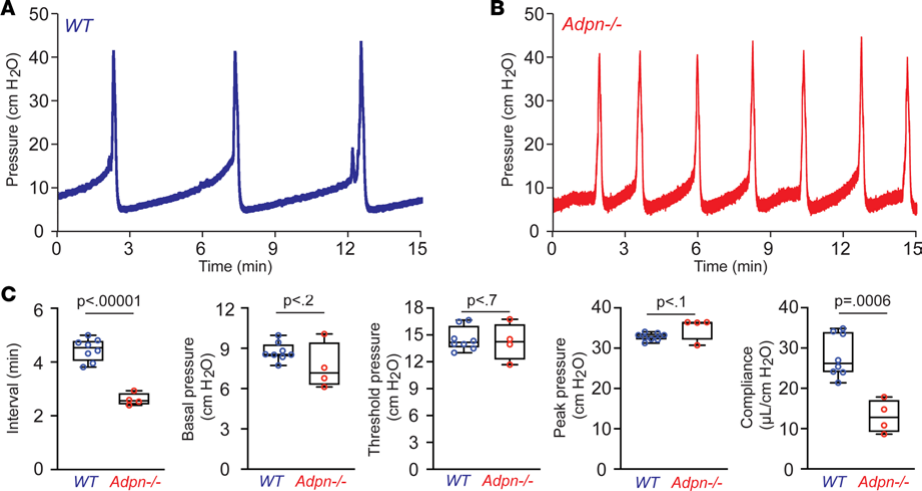
*Adpn^–/–^* mice exhibit voiding frequency, with reduced compliance. Representative CMG traces of (**A**) WT (*n* = 8) and (**B**) *Adpn^–/–^* mice (*n* = 4). (**C**) Quantification of data in **A** and **B**. Data are plotted in box (75% of the data) and whisker format (minimum to maximum), with centerline as median value. Student’s *t* test, with *P* values above bars.

We performed voiding spot assays (VSAs) to examine whether deletion of ADPN can directly cause voiding dysfunction in *Adpn^–/–^* mice (Figure 1). Whereas wild-type (WT) mice produce approximately 3 primary voiding spots (PVS; spot area > 80 mm^2^) over 4 hours (PVS; male: 3.2 PVS/4 hours; female: 3.5 PVS/4 hours), mean PVS number in *Adpn^–/–^* mice was significantly higher (male: 4.9 PVS/4 hours; female: 5.0 PVS/4 hours). In contrast, *Adpn^–/–^* mean PVS volume was significantly smaller (male: 94.2 μL/void; female: 85.1 μL/void) than for WT mice (male: 151.1 μL/void; female: 108.5 μL/void) (Figure 1). To confirm the altered voiding pattern detected in the VSA and to explore potential underlying mechanisms, urodynamic studies were performed by cystometrogram (CMG). CMG data consistently indicated that *Adpn^–/–^* mice exhibited increased voiding frequency with decreased bladder compliance (Figure 2).

### Adpn^–/–^ mice have diminished smooth muscle contractile force.

**Figure 3:**
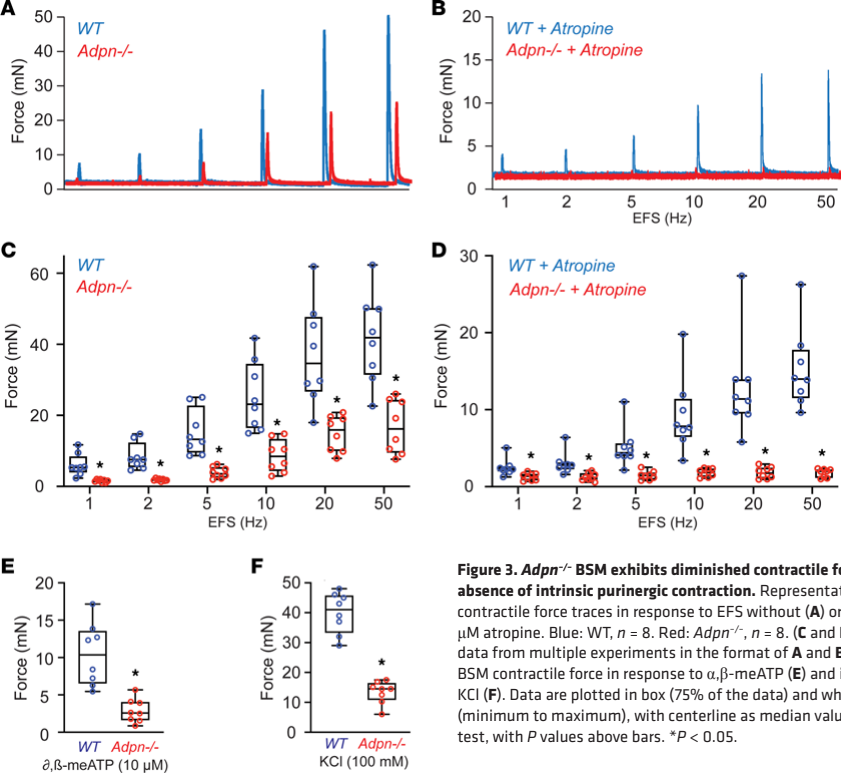
*Adpn^–/–^* BSM exhibits diminished contractile force, with absence of intrinsic purinergic contraction. Representative BSM contractile force traces in response to EFS without (**A**) or with (**B**) 0.5 μM atropine. Blue: WT, *n* = 8. Red: *Adpn^–/–^*, *n* = 8. (**C** and **D**) Summarized data from multiple experiments in the format of **A** and **B**. (**E** and **F**) BSM contractile force in response to α,β-meATP (**E**) and in response to KCl (**F**). Data are plotted in box (75% of the data) and whisker format (minimum to maximum), with centerline as median value. Student’s *t* test, with *P* values above bars. *P < 0.05.

BSM cells play a central role in bladder contraction and relaxation. As our previous study detected ADPN expression in BSM cells (17), we hypothesized that *Adpn* deletion could impair BSM contractility and alter the bladder-voiding phenotype in *Adpn^–/–^* mice (Figures 1 and 2). Myography was performed on isolated BSM strips (Figure 3, A and C). BSM strip contractile force increased in response to increased electrical field stimulation (EFS) frequencies, mimicking the in vivo BSM contraction in response to neurotransmitter release. The contractile force of *Adpn^–/–^* mouse BSM strips was significantly lower than for WT BSM strips (Figure 3, A and C). WT bladder contraction is mainly mediated by parasympathetic ATP and acetylcholine (ACh) co-release, which induces purinergic P2X1 and muscarinic CHRM3 receptor–mediated signaling cascades, leading to BSM contraction (30). To measure possible changes of these signaling pathways in *Adpn^–/–^* mouse BSM, we tested the effect on BSM contraction of the CHRM3 receptor antagonist, atropine. As expected, atropine significantly inhibited WT mouse BSM contractile force in response to EFS (Figure 3, B and D), and the remaining contractile force (~40%) was generally purinergic. To our surprise, atropine completely inhibited *Adpn^–/–^* mouse BSM contraction in response to EFS (Figure 3, B and D), suggesting a lack of purinergic contraction in the *Adpn^–/–^* mouse bladder. Further studies using the P2X1 receptor agonist α,β-methyleneadenosine 5′-triphosphate (α,β-meATP) confirmed minimal purinergic contractile force in the *Adpn^–/–^* mouse bladder (Figure 3E). To confirm that the diminished contractile force in the *Adpn^–/–^* mouse bladder is intrinsic to BSM and not secondary to altered neurotransmitter release, we tested BSM response to depolarization by high extracellular KCl. Results consistently indicated significantly smaller KCl-induced contractile force in BSM strips from *Adpn^–/–^* mouse bladders (Figure 3F).

### ADPN receptor agonist AdipoRon dose-dependently inhibits BSM contractile force.

**Figure 4:**
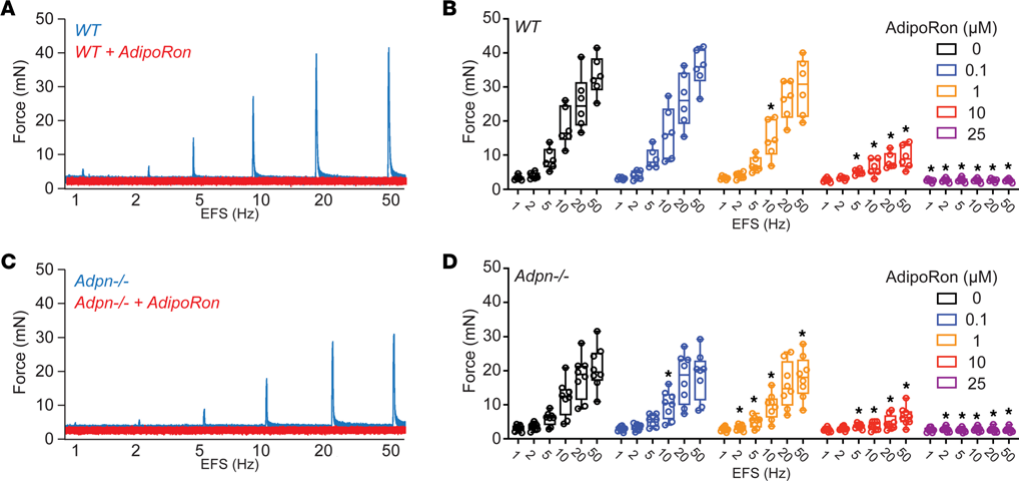
Adiponectin receptor agonist AdipoRon inhibits mouse BSM contractile force in a dose-dependent manner. (**A** and **C**) Representative traces of male WT (*n* = 6) and *Adpn^–/–^* (*n* = 8) BSM contraction in response to EFS before (blue) and after (red) exposure to accumulative addition of AdipoRon. (**B** and **D**) Summarized data from experiments similar to those in **A** and **C**, respectively. Data are plotted in box (75% of the data) and whisker format (minimum to maximum), with centerline as median value. One-way ANOVA was used to examine the differences among groups of specific EFS frequency; data were then compared to control responses with a paired Student’s *t* test to test the effect before and after drug intervention. *P < 0.05.

As *Adpn^–/–^* mouse BSM exhibited profoundly diminished contractile force, we tested whether pharmacological modulation of ADPN signaling could regulate acute BSM contractile force. Interestingly, the selective ADPN receptor agonist AdipoRon (31) dose-dependently inhibited EFS-induced BSM contractile force in both WT and *Adpn^–/–^* mice (Figure 4, A–D). AdipoRon inhibition of BSM contractile force was apparent at approximately 1 μM and complete at approximately 25 μM in both WT and *Adpn^–/–^* mice, indicating intact ADPN receptors in the *Adpn^–/–^* mouse bladder. AdipoRon also dose-dependently inhibited BSM contractile force stimulated by carbachol, by α,β-meATP, and by KCl (Supplemental Figure 2; supplemental material available online with this article; https://doi.org/10.1172/jci.insight.188780DS1). These data reveal a novel function of AdipoRon in regulating acute BSM contractile force.

### AMPK signaling is crucial for BSM purinergic contractile force.

**Figure 5:**
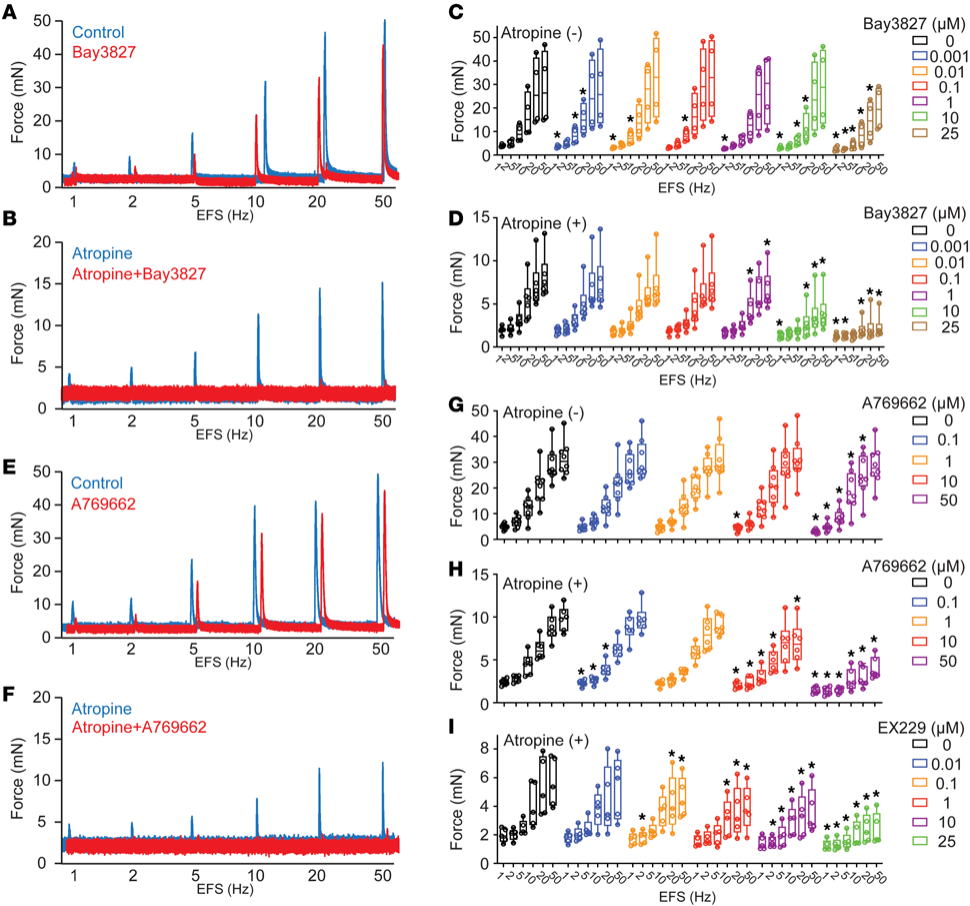
AMPK modulators inhibit mouse BSM purinergic contraction. (**A**) Representative traces of WT BSM contraction in response to EFS (*n* = 4) before (blue) and after (red) exposure to AMPK inhibitor BAY-3827. (**B**) Representative traces of atropine-pretreated WT male BSM contraction in response to EFS (*n* = 6) before (blue) and after (red) exposure to AMPK inhibitor BAY-3827. (**C** and **D**) Summarized data from experiments in the formats of **A** and **B**. (**E**) Representative traces of WT male BSM contraction in response to EFS (*n* = 8) before (blue) and after (bed) exposure to AMPK activator A769662. (**F**) Representative traces of atropine-pretreated WT male BSM contraction in response to EFS (*n* = 6) before (blue) and after (red) exposure to AMPK activator A769662. (**G**–**I**) Summarized data from experiments in the formats of **E** and **F**. Data are plotted in box (75% of the data) and whisker format (minimum to maximum), with centerline as median value. One-way ANOVA was used to examine the differences among groups of specific EFS frequency; data were then compared to control responses with a paired Student’s *t* test to assess the effect before and after drug intervention. *P < 0.05.

AMP-activated protein kinase (AMPK) is an energy sensor that regulates glucose and energy metabolism (32), and a well-known downstream effector of ADPN receptor activation. However, a role for AMPK signaling in ADPN receptor–mediated BSM contraction has not been reported to our knowledge. We examined BSM contractile responses to AMPK activators and inhibitors. As shown in Figure 5, A and C, the selective AMPK inhibitor BAY-3827 partially inhibited EFS-induced BSM contraction. Interestingly, this inhibition seems more sensitive to lower-frequency than to high-frequency EFS-induced BSM contraction (Figure 5C). As BSM purinergic contraction shares higher sensitivity to lower-frequency EFS, whereas high-frequency EFS-induced BSM contractility predominantly reflects muscarinic contraction, we suspected that AMPK inhibitor BAY-3827 might specifically inhibit purinergic contraction of BSM. Indeed, in the presence of atropine to block muscarinic receptors while sparing purinergic signaling (Figure 5B), BAY-3827 inhibited most of the purinergic contractility of BSM (Figure 5, B and D).

We further tested whether AMPK activation could impact acute BSM contractile force. To our surprise, AMPK activation also inhibited the purinergic contraction of BSM. We used the potent AMPK activator A769662 to test this possibility. As did AMPK inhibitor BAY-3827, the potent AMPK activator A769662 partially inhibited EFS-induced BSM contractility, reflecting near-complete inhibition of the purinergic component of BSM contraction (Figure 5, E–H). The additional selective and potent AMPK activator EX229 similarly inhibited purinergic BSM contractility (Figure 5I). These apparently paradoxical pharmacological data suggest that both activators and inhibitors of AMPK can inhibit purinergic contraction of BSM.

### Adpn^–/–^ mouse bladders exhibit altered expression of purinergic signaling molecules.

**Figure 6:**
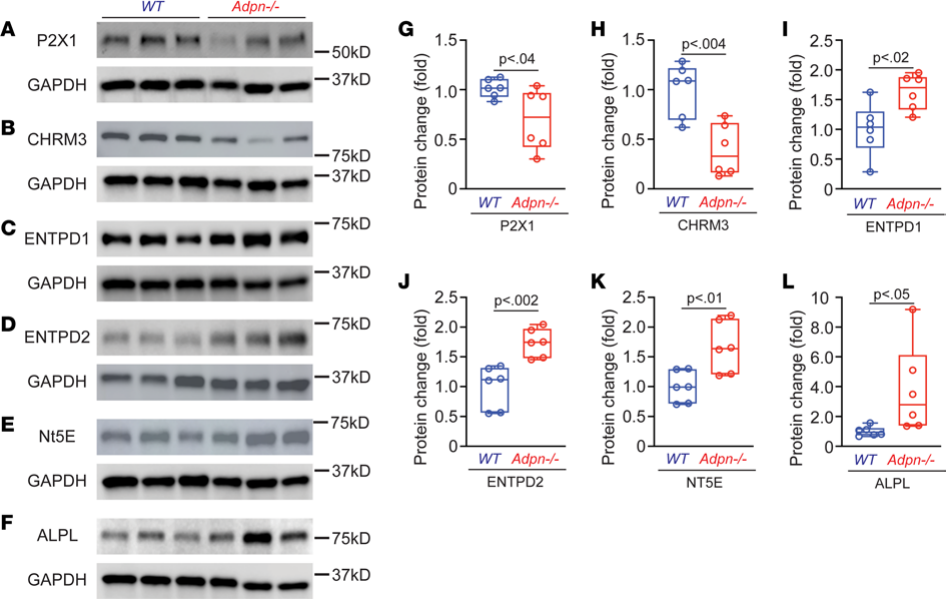
Changes in purinergic signaling components correlate with diminished BSM purinergic contractile force in *Adpn^–/–^* bladders. (**A**–**F**) Western blots of P2X1, CHRM3, ENTPD1, ENTPD2, NT5E, and ALPL proteins from male WT and *Adpn^–/–^* mouse bladders (*n* = 6). (**G**–**L**) Quantitated data normalized to GAPDH are plotted in box (75% of the data) and whisker format (minimum to maximum), with centerline as median value. Student’s *t* test, with *P* values shown above bars. The membrane used for P2X1 detection in **A** was stripped and reblotted for αSMA detection (see Figure 7C), and therefore shares the same loading control.

To further understand the mechanism of impaired smooth muscle contractile function in *Adpn^–/–^* mice, we performed Western blot studies to detect proteins of the signaling pathways regulating BSM contraction. Consistent with our functional data showing diminished EFS-induced BSM contractility (Figure 3), we found expression levels of both CHRM3 receptor and P2X1 receptor to be significantly downregulated in *Adpn^–/–^* BSM (Figure 6, A, B, G, and H). The lack of purinergic contractility in *Adpn^–/–^* BSM prompted further interrogation of other purinergic signaling components in the bladder wall. Interestingly, BSM-expressed ATP-ADP-adenosine–converting enzymes ENTPD1 and NT5E were both significantly upregulated in *Adpn^–/–^* bladders (Figure 6, C, E, I, and K). The interstitium-localized ENTPD2 and urothelium-expressed alkaline phosphatase (ALPL) were also significantly increased (Figure 6, D, F, J, and L) (33, 34). As P2X1 is a major mediator of bladder purinergic contraction, and as ectonucleotidases convert the contractile agonists ATP and ADP into the relaxation agonist adenosine (30, 33, 35, 36), the observed decrease in P2X1 receptor and increase in purine nucleotide phosphatases result in diminished purinergic contractile force in *Adpn^–/–^* bladders.

### Adpn^–/–^ mouse bladders exhibit altered proliferation and differentiation.

**Figure 7:**
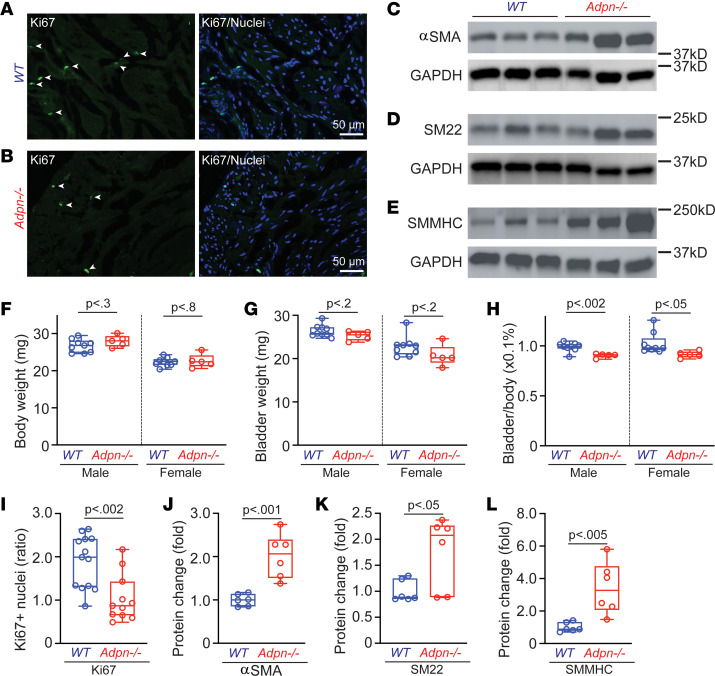
*Adpn^–/–^* BSM cells exhibit altered proliferation and differentiation phenotypes. (**A**) WT (*n* = 4) and (**B**) *Adpn^–/–^* (*n* = 4) bladder tissues immunostained for proliferation marker Ki67 (green, white arrowheads) colocalized with DAPI-stained nuclei (blue). Scale bars: 50 μm. (**C**–**E**) Western blots of smooth muscle markers αSMA, SM22, and SMMHC proteins in WT and *Adpn^–/–^* bladders (*n* = 6). (**F**–**H**) Body weights, bladder weights, and bladder-to-body weight ratios of male and female WT (*n* = 9) and *Adpn^–/–^* (*n* = 5) mice. (**I**) Percentage Ki67-positive cells in BSM layer (WT *n* = 13 sections; *Adpn^–/–^* *n* = 11 sections), defined by number of Ki67-positive nuclei divided by the number of all nuclei in each randomly selected field imaged by ×40 objective. (**J**–**L**) GAPDH-normalized densitometric data from **C**–**E**. Data are plotted in box (75% of the data) and whisker format (minimum to maximum), with centerline as median value. Student’s *t* test, with *P* values shown above bars. The membrane used for SMMHC detection in **E** was stripped and reblotted for INSR detection (see Figure 8A) and therefore shares the same loading control.

ADPN signaling is known to regulate cell phenotype through PPARα and other transcription factors important for cell proliferation and differentiation (27). Although the *Adpn^–/–^* mouse bladder weight did not differ from that of WT controls, the bladder/body weight ratio of the *Adpn^–/–^* bladder was significantly lower than WT (Figure 7, F–H). Correspondingly, the numbers of Ki67-positive nuclei in the BSM layer of *Adpn^–/–^* mouse bladders were significantly lower than those found in WT controls (Figure 7, A, B, and I), suggesting decreased BSM cell proliferation in the *Adpn^–/–^* bladder wall. Intriguingly, several smooth muscle marker proteins were significantly upregulated in *Adpn^–/–^* mouse bladder, including α-smooth muscle actin (αSMA), SM22, and smooth muscle myosin heavy chain (SMMHC), all important for BSM contractile function (Figure 7, C–E and J–L). These upregulated proteins indicate that the altered BSM differentiation/phenotype in the *Adpn^–/–^* bladder may reflect disruption of ADPN signaling.

### Adpn^–/–^ mouse bladders exhibit altered downstream signaling.

**Figure 8:**
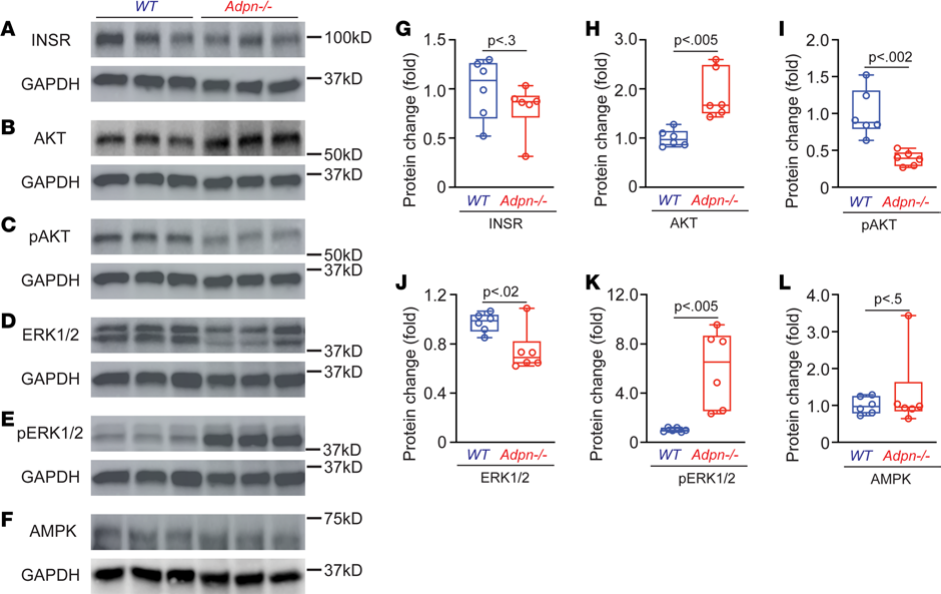
*Adpn^–/–^* BSM cells exhibit altered protein abundance in multiple downstream signaling pathways. (**A**–**F**) Western blot of INSR, AKT, p-AKT, ERK1/2, p-ERK1/2, and AMPK proteins from male WT and *Adpn^–/–^* bladders (*n* = 6). (**G**–**L**) Quantification of the results in **A**–**F**. GAPDH was the normalization control for quantification. Data are plotted in box (75% of the data) and whisker format (minimum to maximum), with centerline as median value. Student’s *t* test, with *P* values shown above bars. The membrane used for AKT (**B**) and p-AKT (**C**) was stripped for reblotting and therefore shares the same loading control. Similarly, ERK1/2 (**D**) and p-ERK1/2 (**E**) have the same loading control due to reblotting.

In addition to its importance in energy and metabolism homeostasis and insulin sensitization, ADPN signaling also regulates gene transcription and protein/lipid synthesis, thereby impacting cell proliferation/differentiation (19, 37). Deletion of ADPN signaling in the *Adpn^–/–^* bladder wall was confirmed by immunofluorescence and Western blotting (Supplemental Figure 1). We have examined additional ADPN-relevant pathways in *Adpn^–/–^* bladders. As shown in Figure 8A, insulin receptor (INSR) expression in *Adpn^–/–^* bladders did not differ from WT (Figure 8G). AKT signaling is a major downstream pathway of INSR activation important for glucose and lipid metabolism. Whereas total AKT in *Adpn^–/–^* bladders increased significantly, p-AKT (p-Ser473) was reduced compared with WT controls (Figure 8, B, C, H, and I). These data thus suggest impaired insulin signaling or potential insulin resistance in the bladder wall of *Adpn^–/–^* mice. Extracellular signal–regulated kinase (ERK) signaling is another pathway downstream of both ADPN receptors and INSRs, important for gene transcription and cell growth (38). *Adpn^–/–^* bladders exhibited decreased total ERK expression, whereas p-ERK (p-Thr202 and p-Tyr204) was increased compared with WT (Figure 8, D, E, J, and K). AMPK expression in the *Adpn^–/–^* bladder did not differ from that in WT (Figure 8, F and L).

## Discussion

The long-recognized clinical association of LUTS and obesity/metabolic syndrome has been little understood. Our study examined the hypothesis that adipokines are major contributors to the pathogenesis of LUTS associated with obesity/metabolic syndrome. We examined in particular the role of ADPN, the most abundant adipokine, in regulation of bladder function. Consistent with our hypothesis, *Adpn^–/–^* mice exhibited significantly increased voiding frequency, small voids, and decreased bladder compliance (Figures 1 and 2). Although peak intravesical pressures did not differ (Figure 2C), the wall tension of the *Adpn^–/–^* mouse bladder, with its smaller voids (and volume), should be greatly reduced per Laplace’s law stating that *T* = *P* × *R*/2 (where *T* is tension, *P* is pressure, and *R* is the radius). Indeed, this is consistent with the significantly decreased BSM contractile force from myography studies in *Adpn^–/–^* mice (Figure 3). Interestingly, lower serum ADPN levels have been significantly associated with LUTS such as detrusor underactivity (DU), defined as insufficient bladder contractile force and decreased voiding efficiency. Lower serum ADPN levels were most strongly associated with decreased maximum flow rate (*Q*_max_), decreased bladder voiding efficiency (BVE), and decreased bladder contractility index (BCI = *P*_det_ @ *Q*_max_ + 5 *Q*_max_; a quantitative value of bladder contraction and ability to sustain that contraction, where *P*_det_ = detrusor pressure). Approximately 75% of patients with DU also exhibited detrusor overactivity (39). Our mouse myography data corroborate this in the significantly diminished contractile force measured in *Adpn^–/–^* bladders (Figure 3, A–C). The bladder phenotype of *Adpn^–/–^* mice combined with the clinically reported inverse association of ADPN with LUTS together support an important role of ADPN in regulation of bladder function. Levels of other adipokines, including nerve growth factor (NGF), interleukins, and TNF-α have also been reported to correlate with OAB (40), suggesting contributions of additional molecular mechanisms to LUTS pathogenesis in association with obesity/metabolic syndrome.

BSM contraction is mediated by both muscarinic and purinergic signaling. We found that, in addition to decreased muscarinic contraction, *Adpn^–/–^* bladders lacked a contractile response to purinergic activation (Figure 3D). The abundance of P2X1, a major purinergic receptor responsible for BSM purinergic contraction, was also significantly decreased in *Adpn^–/–^* bladders (Figure 6, A and G). In contrast, purine-converting and -degrading enzymes in the *Adpn^–/–^* bladder wall, including ENTPD1/2, NT5E, and ALPL, were all upregulated (Figure 6, C–F and I–L). This upregulation is predicted to quickly eliminate purinergic agonists such as ATP and ADP from the *Adpn^–/–^* bladder wall. ATP and ADP stimulation of P2X1 and P2Y12 receptors activates contraction in human and rodent bladders (30). Thus, downregulated purinergic receptor activity combined with upregulation of purine nucleotide–converting and –degrading activities likely contribute to the diminished purinergic contractile force observed in *Adpn^–/–^* bladders.

Bladder contraction is initiated by an ATP-mediated activation of the P2X1 receptor, followed by sustained M3 receptor–mediated contraction to expel urine. The decreased contractile forces often observed in pathological conditions such as obstructive bladder and DBD are usually muscarinic contraction. Maintained purinergic contraction can often compensate for the decrease in muscarinic contractile force. Although the clinical significance of absent purinergic contractile force in the *Adpn^–/–^* bladder remains unknown, it might explain the strong association between lower serum ADPN levels and decreased *Q*_max_, BVE, and BCI in LUTS patients with obesity- and aging-associated DU and OAB (39).

Another interesting finding of our study is the acute effect on BSM contraction by ADPN signaling. However, unlike the diminished contractile force in the *Adpn^–/–^* bladder due to BSM phenotypic changes, the ADPN receptor agonist AdipoRon acutely inhibited BSM contractile force in a dose-dependent manner within 10–15 minutes (Figure 4 and Supplemental Figure 2). This AdipoRon-mediated inhibition applied to all tested contractile stimuli, including EFS, carbachol, α,β-meATP, and KCl (Figure 4 and Supplemental Figure 2). The inhibitory effect was evident at 1 μM and near maximal at 10 μM, concentrations in agreement with the previously reported EC_50_ of AdipoRon (31). Furthermore, AdipoRon dose-dependently inhibited *Adpn^–/–^* mouse BSM contractile force, indicating intact adiponectin receptors in *Adpn^–/–^* BSM (Figure 4, C and D). ADPN signaling exerts an acute vasodilatory effect by relaxing vascular smooth muscle cells (VSMCs) through an undefined mechanism (41–43). ADPN also hyperpolarizes gastric smooth muscle resting membrane potential by increasing K^+^ current, thereby inhibiting excitation-contraction coupling (44). Although the mechanism by which ADPN inhibits acute BSM contraction remains unclear, our observation of acute inhibition of BSM contraction by AdipoRon appears unprecedented in the literature as of the time of writing. ADPN signaling has been reported to activate numerous intracellular signaling pathways, including the phospholipase C/inositol 1,4,5-trisphosphate pathway regulating sarcoplasmic reticulum calcium release in skeletal muscle. A similar pathway mediates muscarinic contractility in BSM cells, but cannot be easily reconciled with our observation that AdipoRon inhibits BSM contraction. A more thorough mechanistic understanding of ADPN action in BSM may require studies of mouse models with selective ADPN receptor subtype knockout. Extensive tests of chronic AdipoRon treatment in animal models have revealed prolongation of lifespan, with beneficial effects on metabolic syndrome, chronic inflammation, and age-related dysfunctions such as neuronal degeneration and muscle loss (45, 46). Our observation of AdipoRon’s acute effect on BSM contractility suggests an application of therapeutic inhibition of ADPN signaling in BSM to treat LUTS patients, meriting further investigation.

Interestingly, pharmacological inhibition of AMPK signaling specifically blocked acute purinergic contraction of BSM (Figure 5, A–D), consistent with the diminished purinergic contractility observed in *Adpn^–/–^* BSM (Figure 3D). Paradoxically, pharmacological activation of AMPK signaling also acutely blocked the purinergic contraction of BSM (Figure 5, E–I). This latter unexpected finding was consistent with reported ADPN effects on vasodilation and gastric smooth muscle relaxation (41, 44), and with our AdipoRon data (Figure 4 and Supplemental Figure 2). AMPK is an energy-sensing master kinase known to modulate the activity of more than 100 proteins. AMPK activation by low-potency agonist 5-aminoimidazole-carboxamide ribonucleoside (AICAR) partially inhibited aortic smooth muscle contraction (47). AMPK signaling has also been shown to inhibit tonic vascular smooth muscle contraction by direct AMPK phosphorylation of myosin light chain kinase (MLCK) at Ser85, preventing MLCK activation by blocking its calmodulin binding site (48). Perhaps AMPK regulates a finely balanced energy state required to achieve maximal contractile force in response to acute stimulation by neurotransmitter and autocrine agonists. This balanced resting state may reflect the well-known optimized prestretched smooth muscle length required to generate subsequently stimulated maximal contractile force, such that either overstretched or relaxed basal states will generate reduced contractile force (49).

ADPN signaling has been shown to regulate both proliferation and differentiation of VSMCs, each important for the maintenance of normal VSMC contractile phenotype (50–52). The reduced cell proliferation in the *Adpn^–/–^* bladder (Figure 7) might reflect decreased p-AKT signaling (Figure 8C). In contrast, smooth muscle biomarkers αSMA, SM22, and SMMHC (Figure 7) were all increased in abundance, with correspondingly increased p-ERK (Figure 8E). We speculate that reduced proliferation in the *Adpn^–/–^* bladder (Figure 7, A, B, and F–J) may reflect a hypertrophic response mediated through p-ERK signaling to compensate for deficient BSM contractile function (38). The data together indicate considerable complexity of ADPN signaling in BSM.

Our study has several important limitations. Although circulating ADPN derives mainly from adipocytes, BSM also expresses ADPN locally (17). The relative importance or differential functions of adipocyte-derived and BSM-expressed ADPN in regulating bladder function have not been addressed. The identities of the specific ADPN receptors involved and details of receptor-specific downstream signaling pathways are yet to be studied, although single-cell sequencing data indicate the expression of both ADPN receptors 1 and 2 in BSM cells (53). Smooth muscle–specific ADPN receptor–knockout animal models are currently under development in our laboratory to further address these central questions.

In summary, we have described a bladder phenotype in *Adpn^–/–^* mice that recapitulates symptoms and urodynamics observed in humans with LUTS associated with obesity/metabolic syndrome. We have also demonstrated that ADPN signaling plays a profound role in regulating BSM contractility and urodynamics. Disruption of ADPN signaling leads to both metabolic and purinergic signaling pathway changes in the bladder, which we propose may play important roles in the pathogenesis of LUTS associated with obesity/metabolic syndrome.

## Methods

### Sex as a biological variable.

Our study examined male and female animals, and similar findings are reported for both sexes.

### Reagents.

Unless otherwise specified, all chemicals were obtained from MilliporeSigma and were of reagent grade or better. P2X1 receptor agonist α,β-meATP (catalog 3209), muscarinic receptor agonist carbachol (catalog 2810), and ADPN receptor agonist AdipoRon (catalog 5096) were purchased from R&D Systems. AMPK inhibitor BAY-3827 (catalog HY-112083), AMPK activator A769662 (catalog HY-50662), and AMPK activator EX229 (catalog HY-112769) were purchased from MedChemExpress.

### Animals.

All mice used in this study were of the C57BL/6J background and matched for sex and age (12–16 weeks). Both male and female mice were used unless otherwise specified. Mice were housed in standard polycarbonate cages with free access to normal food and water. WT C57BL/6J mice and *Adpn^–/–^* mice (stock 008195) were purchased from The Jackson Laboratory.

### VSA.

VSAs were performed during the daytime, with lights on from approximately 9 am to 1 pm for both male and female mice. Individual mice were gently placed in a standard mouse cage lined with Blicks Cosmos Blotting Paper (catalog 10422-1005), without drinking water but with standard dry mouse chow available. Blotting paper was recovered and imaged as described previously (54). Overlapping voiding spots were visually examined and manually separated by outlining and copying, and then pasting into a nearby empty space in Fiji software (55) for analysis. A volume/area standard curve defined a 1-mm^2^ voiding spot as representing 0.283 μL of urine. Urine spots with an area of 80 mm^2^ or greater were considered PVS based on a cutoff established from the voiding spot patterns of hundreds of mice (54, 56).

### CMG.

CMG performed in WT female mice with PBS infusion (25 μL/min) as previously described produces a consistent voiding pattern (30, 55). With urethane (1.4 g/kg body weight) and continuous-flow isoflurane (3% induction, 1.0% maintenance) anesthetization, a 1-cm midline abdominal incision was performed. PE50 tubing was then implanted through the dome of the bladder. The catheter was connected to a pressure transducer and a syringe pump was coupled to data-acquisition devices (WPI Transbridge and AD Instruments PowerLab 4/35) and a computerized recording system (AD Instruments LabChart software). Isoflurane was withdrawn immediately after surgery and CMG was performed under urethane anesthesia, which spares the voiding reflex. CMG was performed for more than 1 hour of stable, regular voiding cycles. At least 5 cycles of filling and voiding traces were assessed to evaluate urodynamics. Bladder compliance was determined by Δvolume/Δpressure = (Δ*t* × *v*)/Δpressure, where *v* = 25 μL/min.

### BSM myography.

BSM myography was performed on male mice, whose approximately 30-mg bladders (larger than in females) facilitate dissection of the bladder epithelial layer away from BSM for a consistent per-bladder yield of approximately 7-mm by 2-mm muscle strips free of mucosa. Muscle strips were mounted in an SI-MB4 tissue bath system (World Precision Instruments). Force sensors were connected to a TBM 4M transbridge (World Precision Instruments). Signals were amplified by PowerLab (AD Instruments) and monitored with Chart software (AD Instruments). BSM strips gently stretched to optimize contractile force were then pre-equilibrated for at least 1 hour. Contraction force was sampled at 2000/s using Chart software. EFS was carried out with a Grass S48 field stimulator (Grass Technologies), using previously described standard protocols: 50 V, 0.05 ms duration, 3-second trains of stimuli, and frequencies of 1, 2, 5, 10, 20, and 50 Hz (30, 55).

### Immunofluorescent staining and imaging.

Excised bladders fixed in 4% (w/v) paraformaldehyde were cryoprotected, frozen, sectioned, and incubated overnight at 4°C with antibodies (diluted 1:100; Supplemental Table 1). The sections were then incubated with an Alexa Fluor 488–conjugated secondary antibody (diluted 1:100), and nuclei were stained with DAPI. Imaging was performed on an Olympus BX60 fluorescence microscope with a 40×/0.75 NA objective (55). To analyze the proportion of Ki67-positive nuclei in mouse BSM, bladder sections were prepared and immunofluorescent staining was conducted on 4 WT and 4 *Adpn^–/–^* mice. For each mouse bladder tissue, 3–4 images were randomly collected from the Ki67-stained area. The number of Ki67-positive nuclei on the BSM and the total number of nuclei in each image were counted. The ratio of the number of Ki67-positive nuclei to the total number of nuclei in the image was used to determine the Ki67-positive ratio in the BSM. A total of 13 images were analyzed in the WT group and 11 images in the *Adpn^–/–^* group.

### Western blot.

Whole bladder tissues from female mice were lysed with RIPA buffer (50 mmol/L Tris-HCl, 150 mmol/L NaCl, 1% NP-40, 0.5% sodium deoxycholate, and 0.1% SDS). Protein concentrations were determined by BCA protein assay (Thermo Fisher Scientific). Protein (25 μg per well) was fractionated by SDS-PAGE and transferred to PVDF membranes. Membranes were incubated with primary antibodies (Supplemental Table 1) at 4°C for 12 hours. Immunoreactive protein bands were visualized with Amersham ECL reagent. The membranes were incubated with Restore Plus Western Blot Stripping buffer (Thermo Fisher Scientific) for 5 minutes to remove primary and secondary antibodies for reblotting with new antibodies, and each membrane was stripped and reprobed for no more than 3 times. Scanned protein band intensity was quantitated using Fiji software (55). The intensity of the protein band of interest was normalized to glyceraldehyde 3-phosphate dehydrogenase (GAPDH) intensity in the same lane. The average protein expression level in WT mice was designated as 1, and the fold change in protein expression level in knockout mice is reported.

### Statistics.

All data are presented as box-and-whisker plots or as individual symbols and line plots. The centerline in box-and-whisker plots is the median, the box represents 75% of the data, and the whiskers indicate minimum to maximum. Data were analyzed by 1-way ANOVA for comparison among groups. Data were analyzed by 2-tailed Student’s *t* test between 2 groups. If possible, a paired *t* test was used. Bonferroni’s multiple-comparison post hoc test was used where necessary, and a *P* value of less than 0.05 was considered significant.

### Study approval.

All animal studies were performed in adherence to US NIH *Guide for the Care and Use of Laboratory Animals* (National Academies Press, 2011), and with approval of the Beth Israel Deaconess Medical Center Institutional Animal Care and Use Committee (protocol 007-2022).

### Data and resource availability.

Further information, reagents, and other supporting data in this study are available from the corresponding author upon request. Raw data are available in the supplemental Supporting Data Values file.

## Author contributions

WY conceived and supervised the project, performed cystometrograms and myography, analyzed data, and wrote the manuscript. ZL performed histological and immunostaining and imaging, Western blotting, and analyzed data. AW performed voiding spot assays. SR and SLA contributed to the conception and interpretation of data, and revised the manuscript. All coauthors critically reviewed the manuscript, discussed ideas and results, and contributed to the manuscript. WY is the guarantor of this work and, as such, has full access to all the data in the study and takes responsibility for the integrity of the data and the accuracy of the data analysis.

## Supplementary Material

Supplemental data

Unedited blot and gel images

Supporting data values

## Version 1 02/24/2025

Electronic publication
