# Supplementary material for: Adiponectin signaling regulates urinary bladder function by blunting smooth muscle purinergic contractility
Source: JCI Insight. 2025 Feb 24;10(4):e188780. doi: 10.1172/jci.insight.188780 (PMC11949013; doi:10.1172/jci.insight.188780)
Supplement: Supplemental data [file jciinsight-10-188780-s116.pdf]

Supplemental information

Adiponectin Signaling Regulates Urinary Bladder Function by Blunting Smooth Muscle Purinergic Contractility

Zhaobo Luo,[1] Ali Wu,[1] Simon Robson,[3,5] Seth L. Alper,[1,2,4] and Weiqun Yu[1,2,4]*

[1]Division of Nephrology and Departments of [2]Medicine and [3]Anesthesia, Beth Israel Deaconess Medical Center, Boston, MA 02215; [2]Departments of [4]Medicine and [5]Anesthesia, Harvard Medical School, Boston, Massachusetts 02115

Supplemental Figure 1. Adiponectin protein is absent from $Adpn^{-/-}$ mice BSM cells. **A**: *Wild-type*) and **B**: $Adpn^{-/-}$ bladder tissues immunostained with anti-adiponectin antibody (green) and anti-P2X1 antibody (red). ADPN is localized in BSM layer (**A**) and completely absent from $Adpn^{-/-}$ bladder tissue (**B**). The P2X1 signal is significantly decreased in $Adpn^{-/-}$ mice BSM cells. Nuclei were labeled with DAPI (blue). U: urothelial cells; L: lamina propria; M: smooth muscle; V: blood vessel. **C**: Western blot of adiponectin protein detected a specific band above 25KD in *wild-type* bladder absent from $Adpn^{-/-}$ bladder (n=6), with GAPDH loading control (n=6).

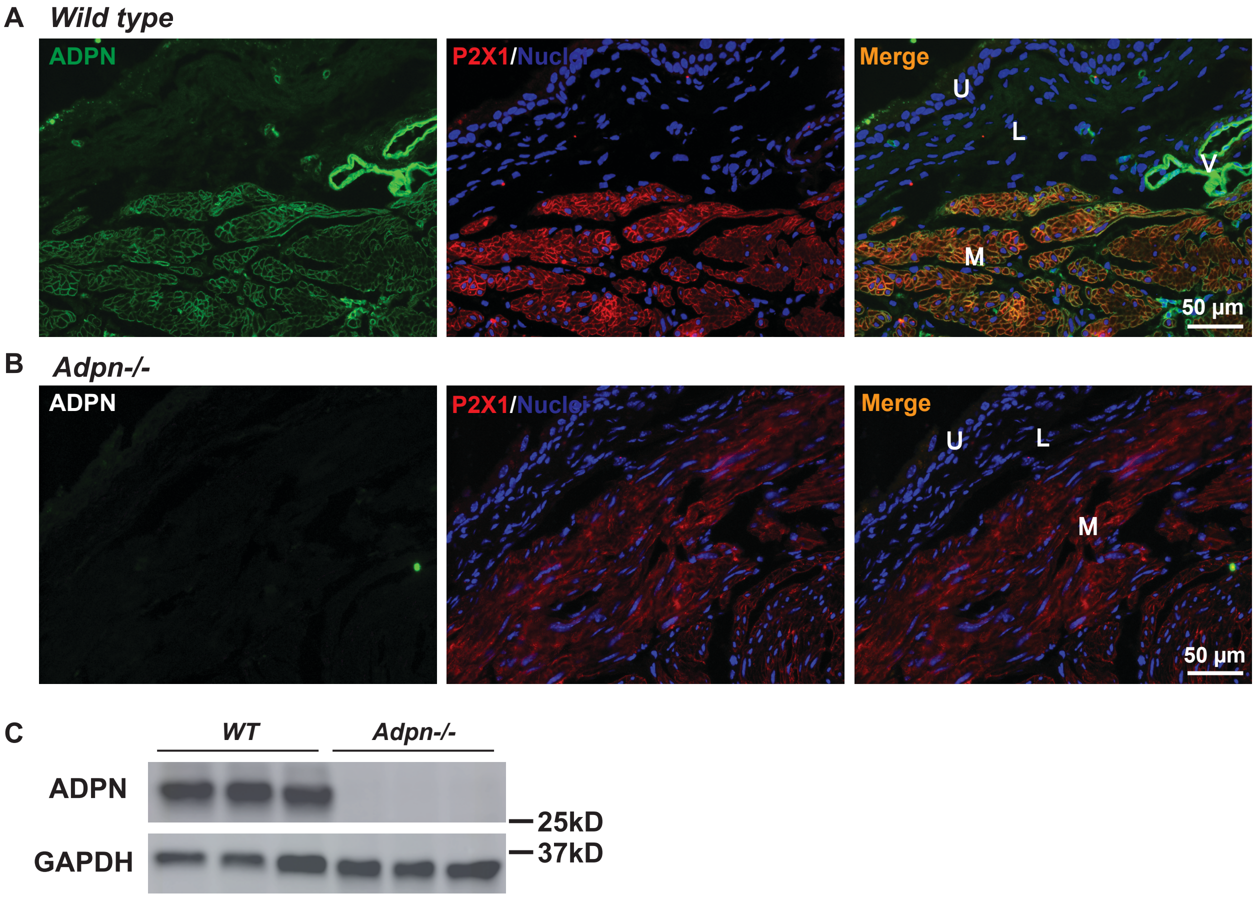

Supplemental Figure 2. Adiponectin receptor agonist AdipoRon inhibited mouse BSM contractile force in a dose-dependent manner. Representative *wild-type* male BSM contraction traces in response to carbachol (**A**), α, β-meATP (**C**), and KCl (**E**), before and after exposure to AdipoRon. **B**, **D**, and **F**: Summarized data corresponding to experiments in formats of panels **A**, **C** and **E** (n=4-8). Data show individual symbols and line plots for each sample. Paired student *t*-test, *P* values above data points. *, $P<0.05$.

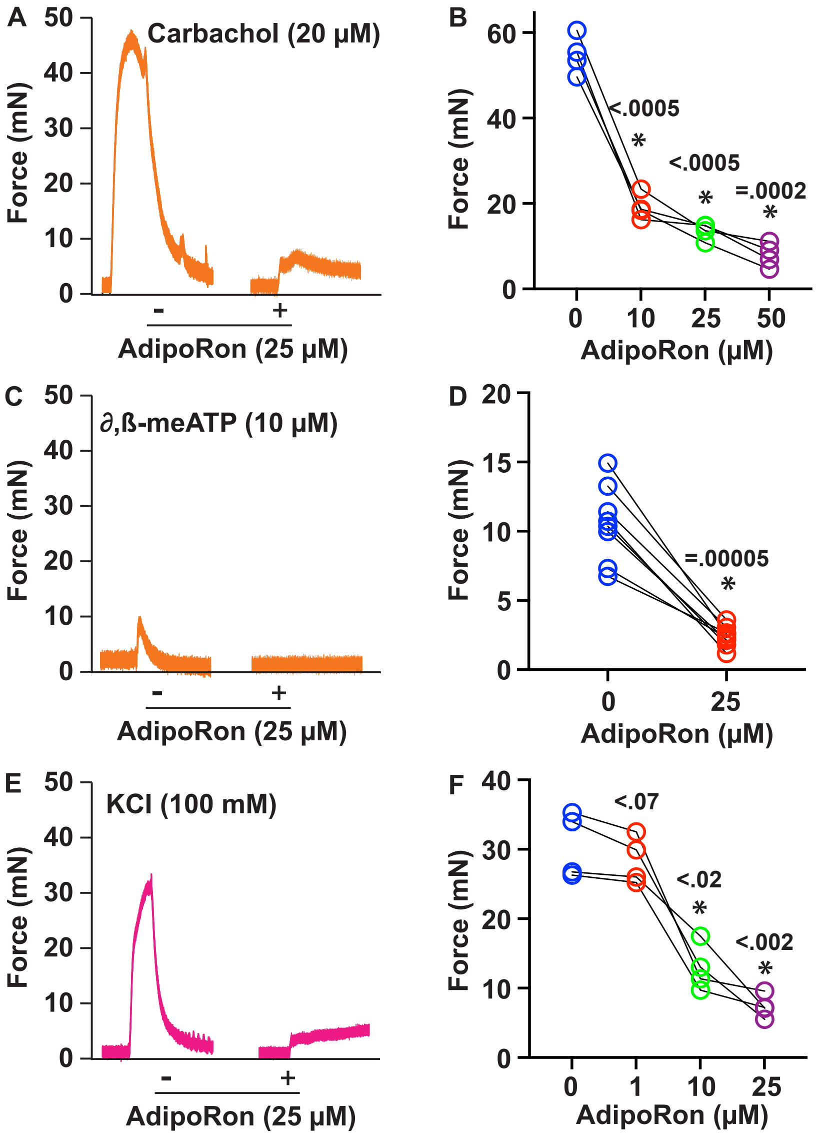

Supplemental Table 1. Antibody information

Antibody	Company	Catalog	Host	Application
Adiponectin	R&D system	AF1119	Goat	WB, IF
Ki67	Invitrogen	14-5698-82	Rat	IF
Alkaline Phosphatase	R&D system	AF2910	Goat	WB
ENTPD1	Invitrogen	MA5-32707	Rabbit	WB
ENTPD2	R&D system	AF5797	Sheep	WB
CHRM3	Invitrogen	PA585322	Rabbit	WB
P2X1	Alomone laboratory	APR-001	Rabbit	WB, IF
αSMA	Cell Signaling Technology	19245S	Rabbit	WB
TAGLN	Cell Signaling Technology	40471S	Rabbit	WB
SMMHC	Proteintech	21404-1-AP	Rabbit	WB
5'-Nucleotidase	R&D system	MAB44881	Rat	WB
Insulin Receptor β	Cell Signaling Technology	23413S	Rabbit	WB
p-AKT	Cell Signaling Technology	9271T	Rabbit	WB
AKT	Cell Signaling Technology	9272S	Rabbit	WB
p-Erk1/2	Cell Signaling Technology	9101S	Rabbit	WB
Erk1/2	ABclonal	A4782	Rabbit	WB
AMPK-α	Cell Signaling Technology	2532S	Rabbit	WB
GAPDH	ABclonal	A19056	Rabbit	WB
