# Supplementary material for: Adiponectin signaling regulates urinary bladder function by blunting smooth muscle purinergic contractility
Source: JCI Insight. 2025 Feb 24;10(4):e188780. doi: 10.1172/jci.insight.188780 (PMC11949013; doi:10.1172/jci.insight.188780)

Full unedited gel for Figure 6. A

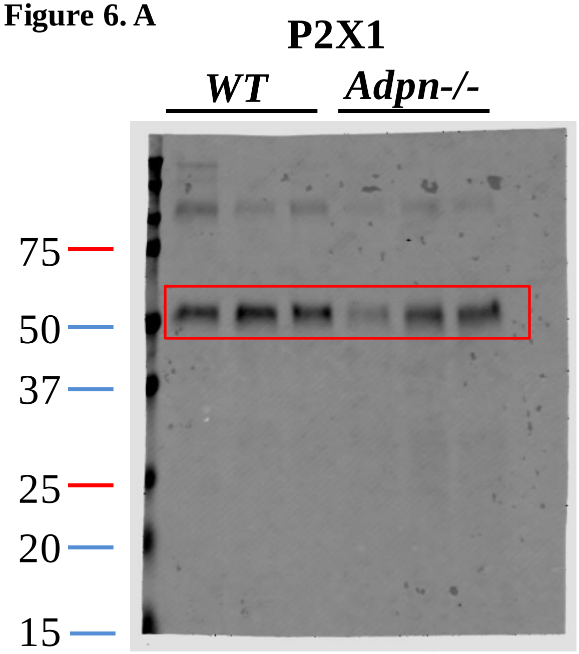

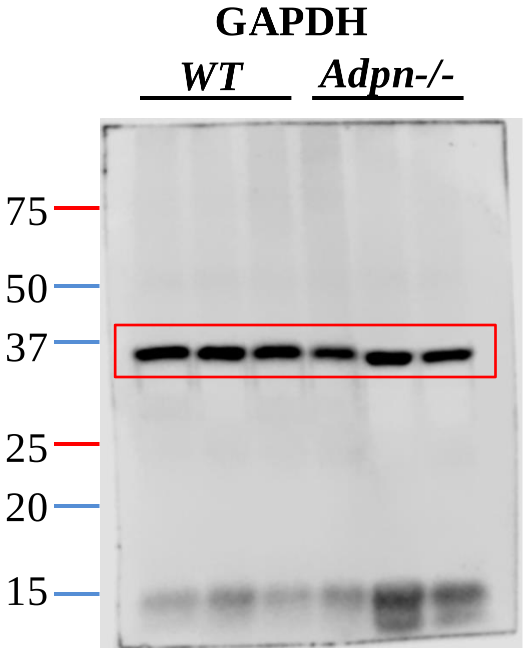

Full unedited gel for Figure 6. B

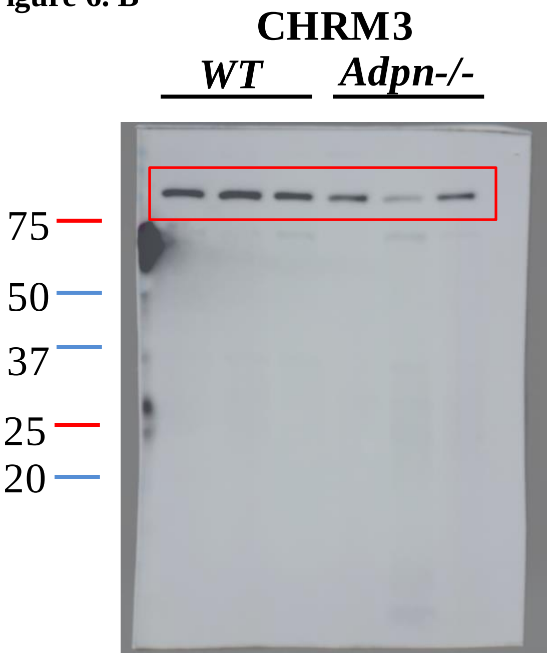

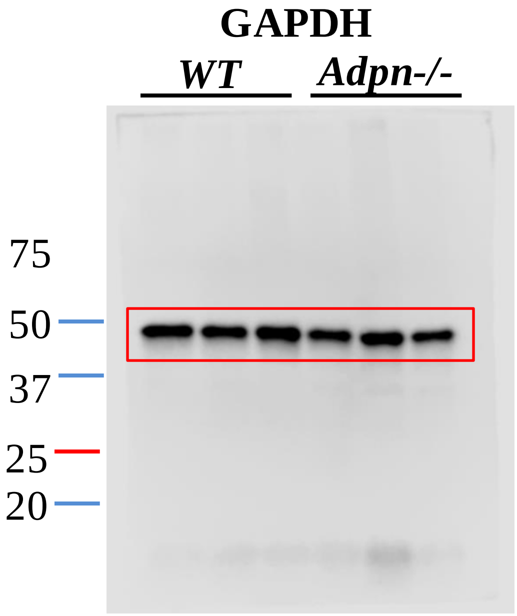

Full unedited gel for Figure 6. C

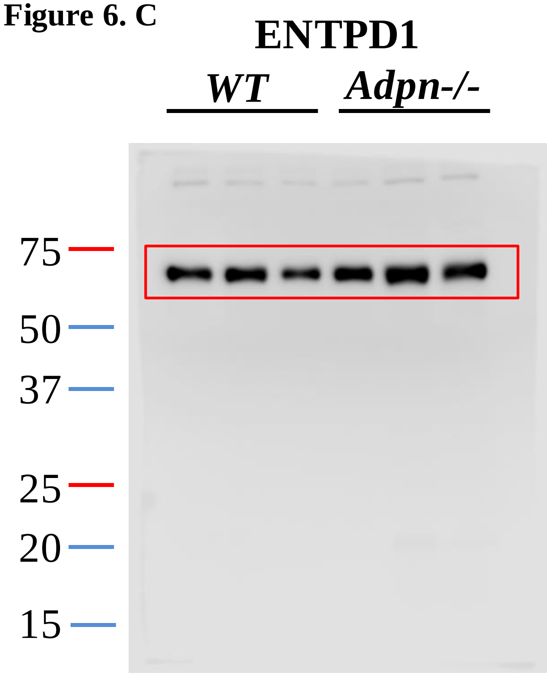

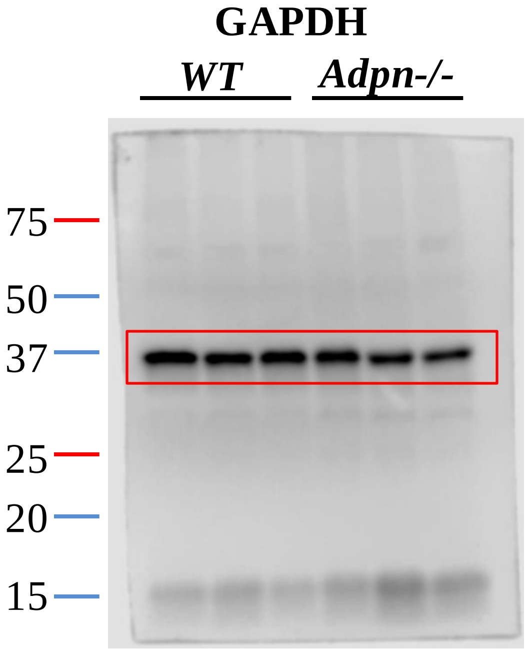

Full unedited gel for Figure 6. D

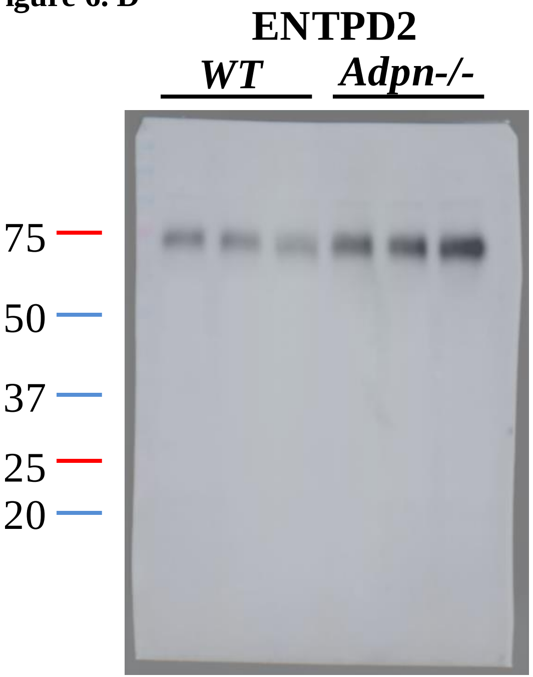

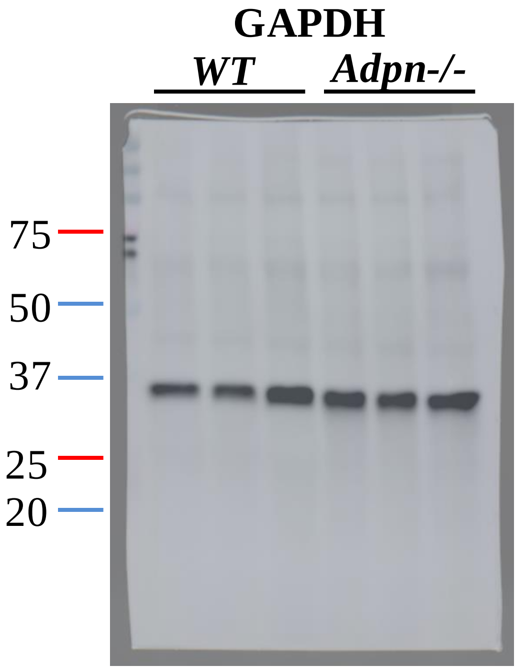

Full unedited gel for Figure 6. E

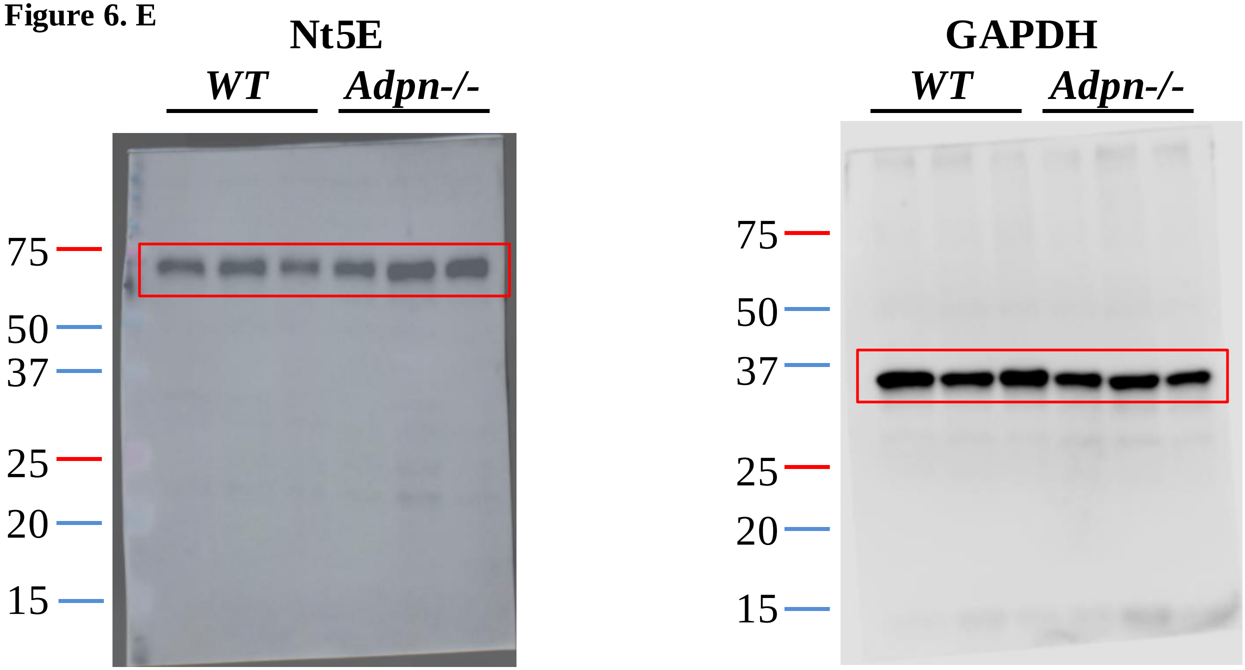

Full unedited gel for Figure 6. F

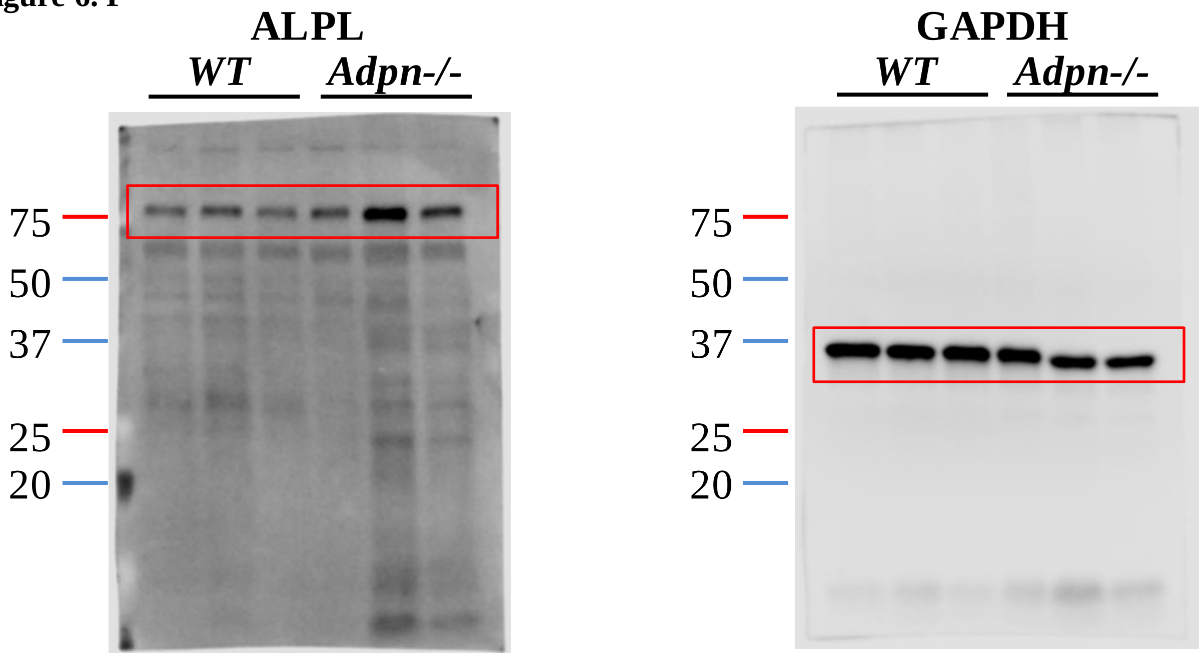

Full unedited gel for Figure 7. C

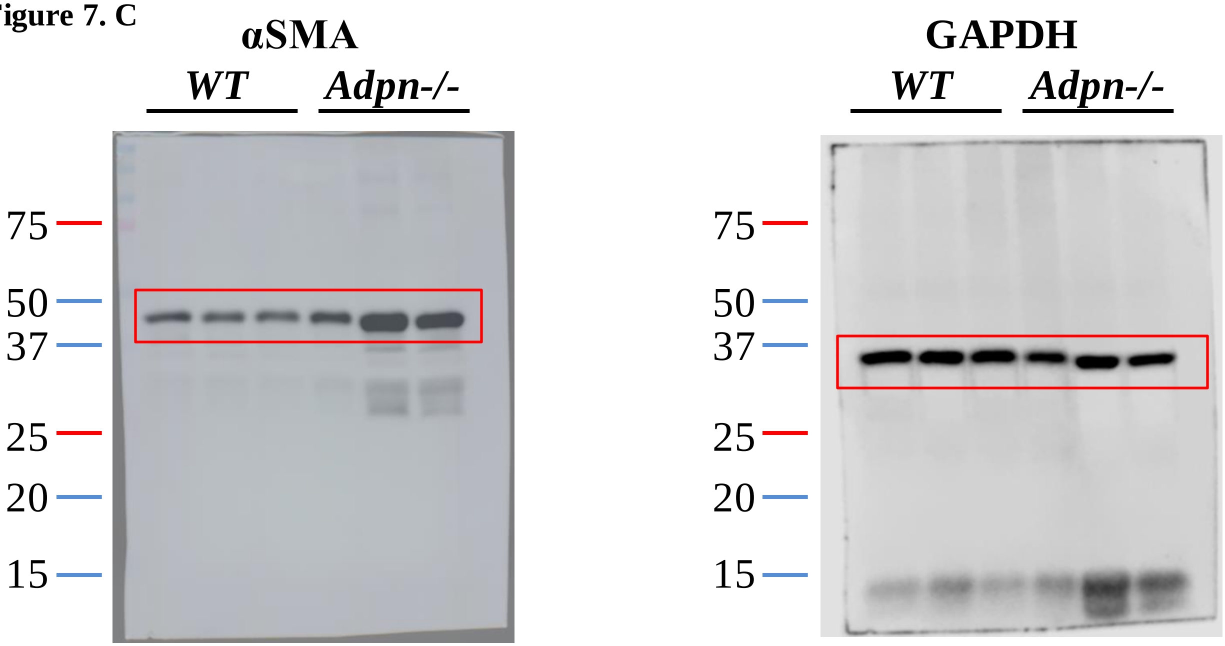

Full unedited gel for Figure 7. D

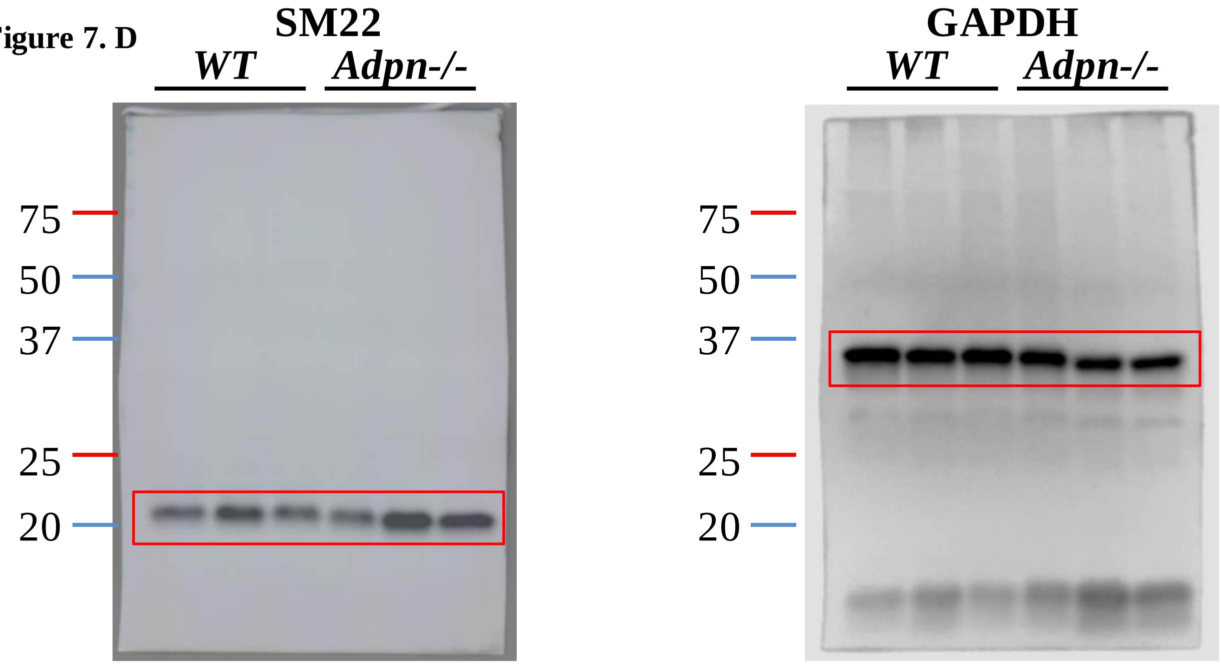

Full unedited gel for Figure 7. E

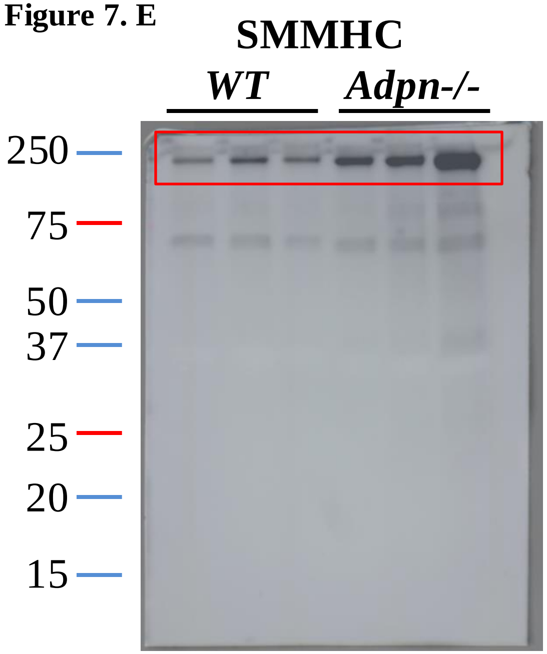

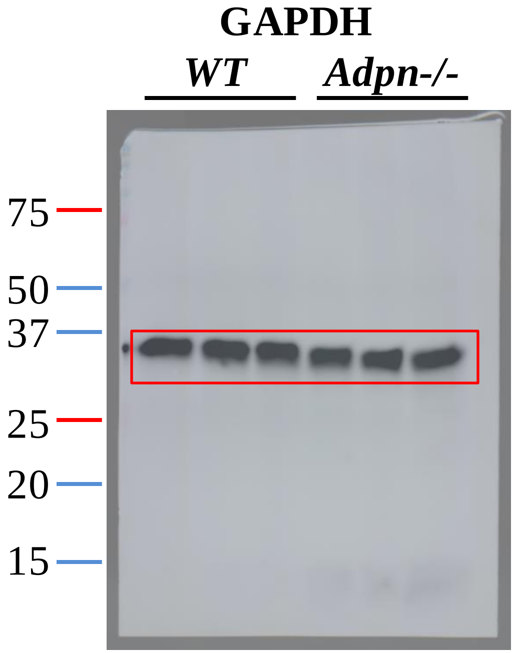

Full unedited gel for Figure 8. A

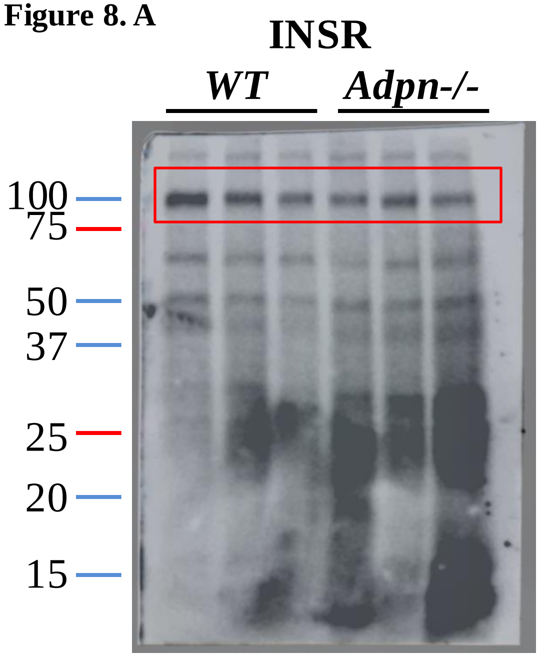

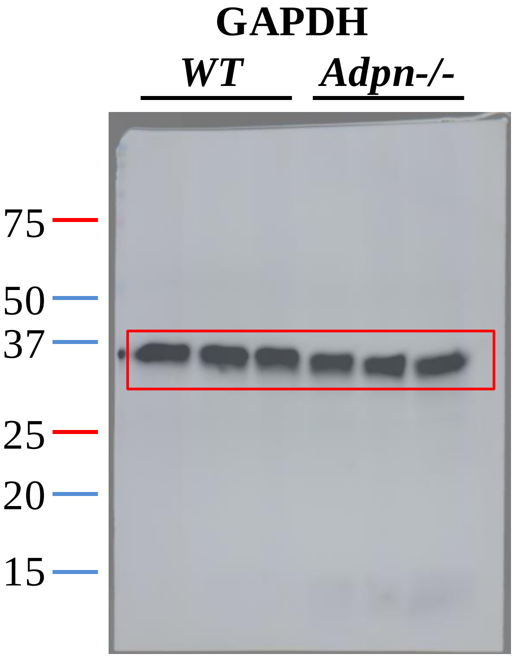

Full unedited gel for Figure 8. B

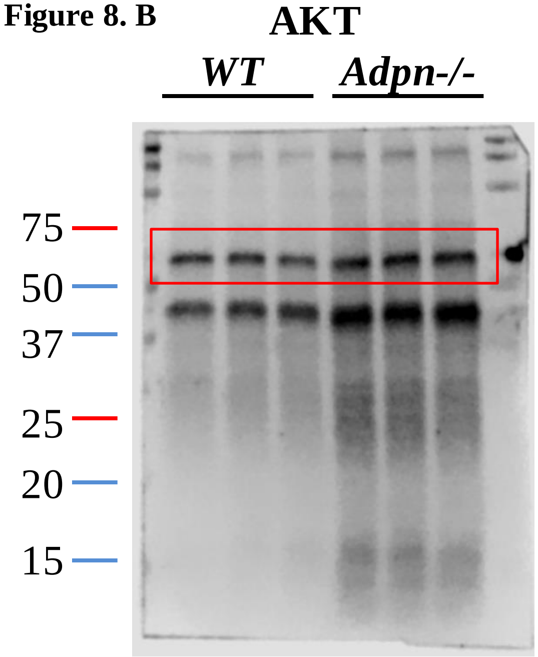

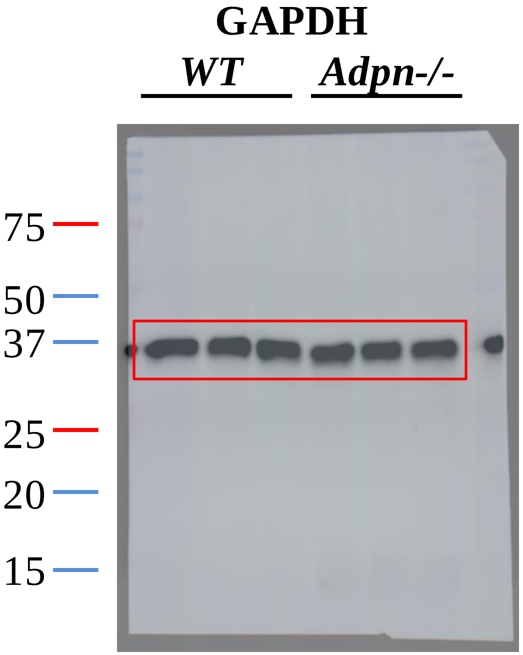

Full unedited gel for Figure 8. C

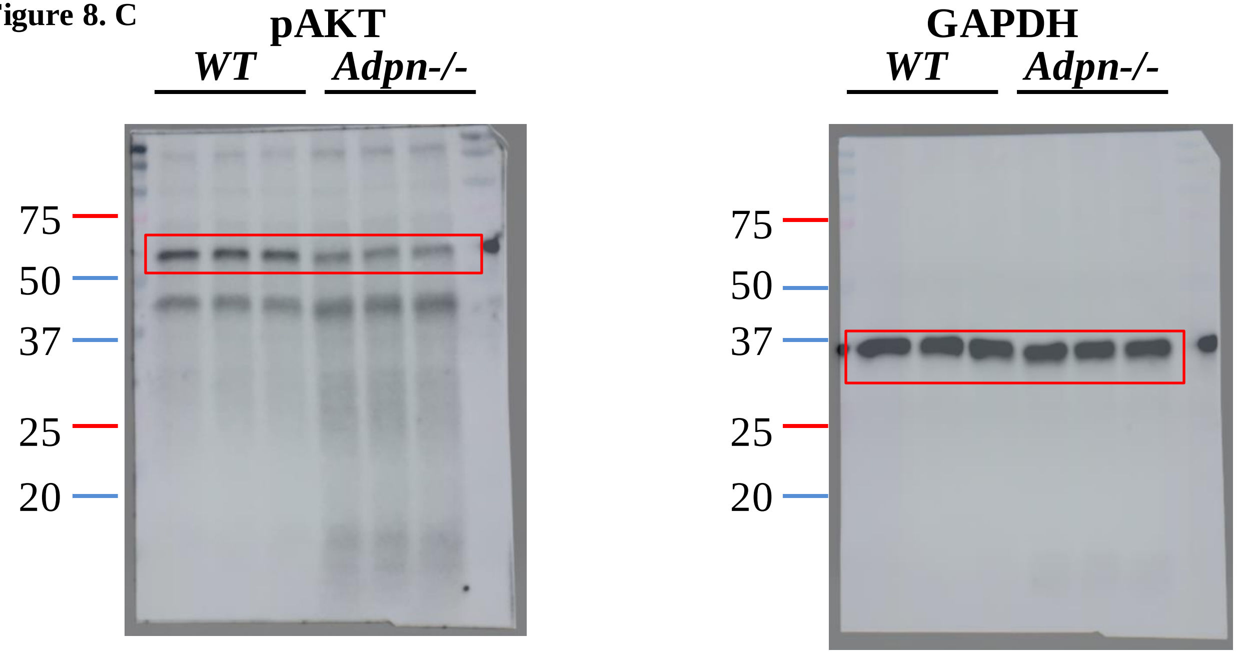

Full unedited gel for Figure 8. D

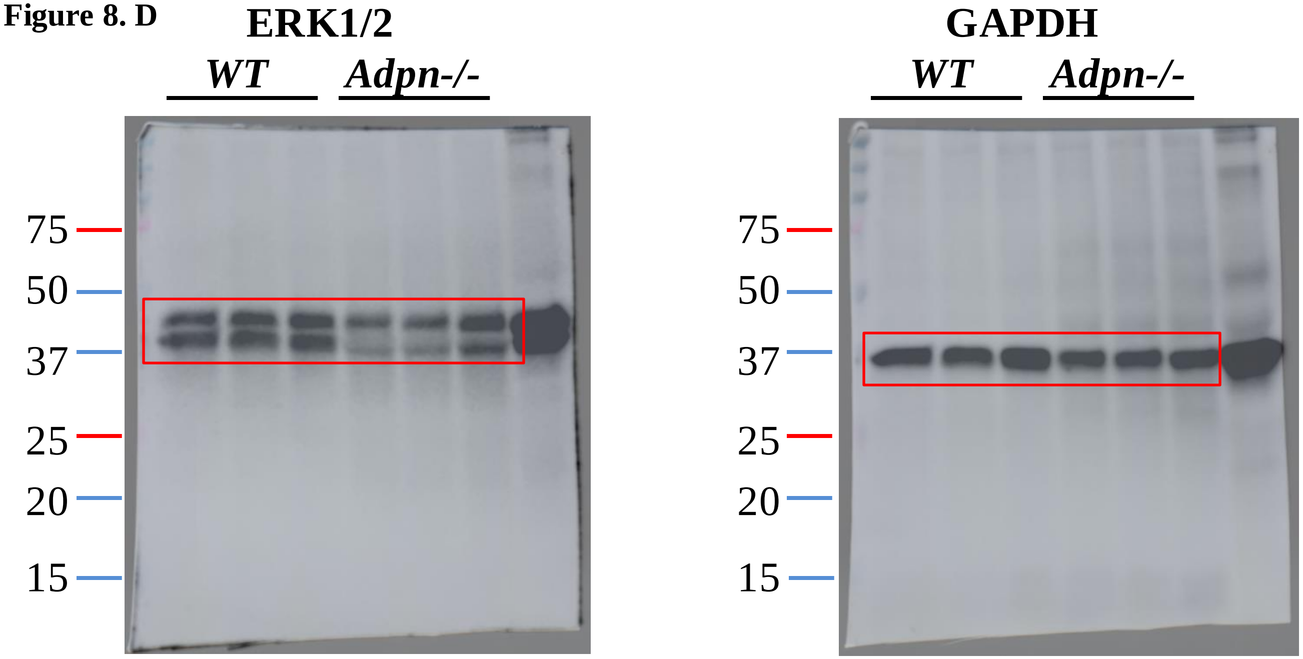

Full unedited gel for Figure 8. E

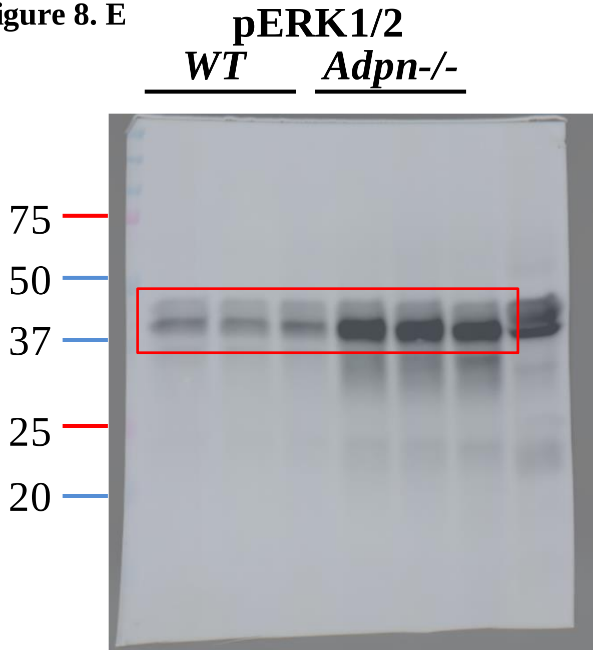

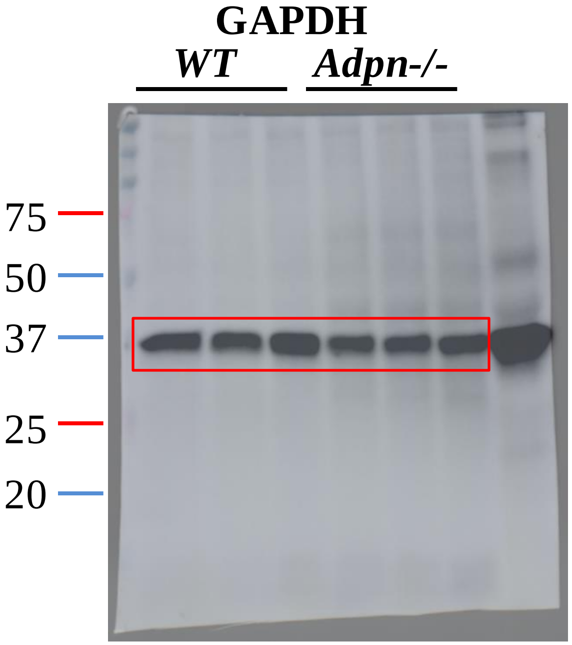

Full unedited gel for Figure 8. F

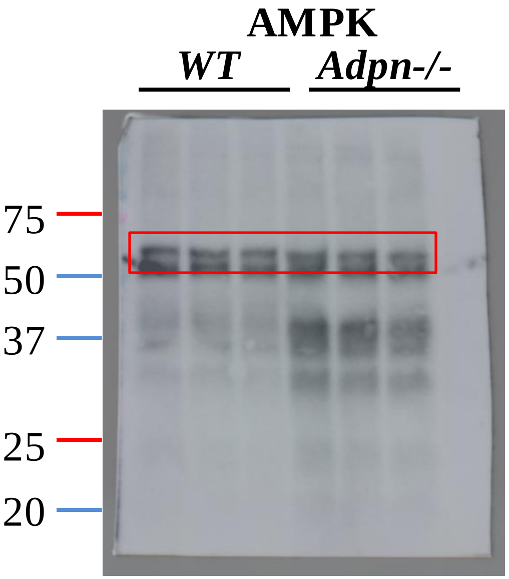

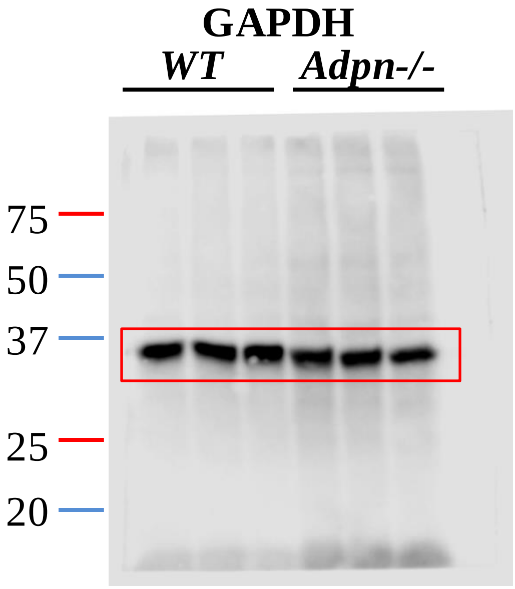

S Figure 1

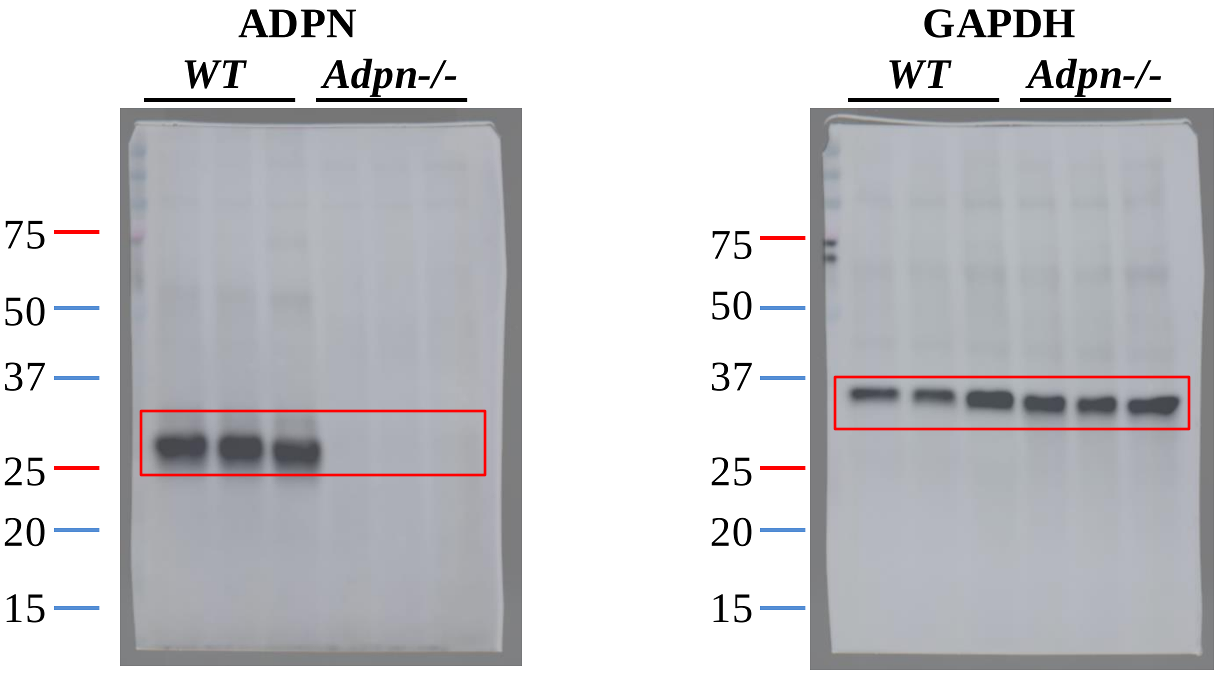

Supplement: Unedited blot and gel images [file jciinsight-10-188780-s117.pdf]
